# Multigenic DNA vaccine induces protective cross-reactive T cell responses against heterologous influenza virus in nonhuman primates

**DOI:** 10.1371/journal.pone.0189780

**Published:** 2017-12-21

**Authors:** Merika T. Koday, Jolie A. Leonard, Paul Munson, Adriana Forero, Michael Koday, Debra L. Bratt, James T. Fuller, Robert Murnane, Shulin Qin, Todd A. Reinhart, Karen Duus, Ilhem Messaoudi, Amy L. Hartman, Kelly Stefano-Cole, Juliet Morrison, Michael G. Katze, Deborah Heydenburg Fuller

**Affiliations:** 1 Department of Microbiology, University of Washington, Seattle, WA, United States of America; 2 Washington National Primate Research Center, University of Washington, Seattle, WA, United States of America; 3 Department of Infectious Diseases and Microbiology, Graduate School of Public Health, University of Pittsburgh, Pittsburgh, PA, United States of America; 4 Center for Immunology and Microbial Disease, Albany Medical College, Albany, NY, United States of America; 5 Basic Sciences Department, College of Osteopathic Medicine, Touro University Nevada, Henderson, NV, United States of America; 6 Division of Pathobiology and Immunology, Oregon National Primate Research Center, Beaverton, OR, United States of America; Icahn School of Medicine at Mount Sinai, UNITED STATES

## Abstract

Recent avian and swine-origin influenza virus outbreaks illustrate the ongoing threat of influenza pandemics. We investigated immunogenicity and protective efficacy of a multi-antigen (MA) universal influenza DNA vaccine consisting of HA, M2, and NP antigens in cynomolgus macaques. Following challenge with a heterologous pandemic H1N1 strain, vaccinated animals exhibited significantly lower viral loads and more rapid viral clearance when compared to unvaccinated controls. The MA DNA vaccine induced robust serum and mucosal antibody responses but these high antibody titers were not broadly neutralizing. In contrast, the vaccine induced broadly-reactive NP specific T cell responses that cross-reacted with the challenge virus and inversely correlated with lower viral loads and inflammation. These results demonstrate that a MA DNA vaccine that induces strong cross-reactive T cell responses can, independent of neutralizing antibody, mediate significant cross-protection in a nonhuman primate model and further supports development as an effective approach to induce broad protection against circulating and emerging influenza strains.

## Introduction

Influenza is a serious public health issue, and new vaccines are needed to better combat seasonal and pandemic strains. The seasonal vaccine relies primarily on antibody responses against hemagglutinin (HA) for protection. The currently licensed live-attenuated and inactivated vaccines induce strong HA-specific antibody and afford significant protection against matched circulating influenza strains however they require annual reformulations to keep pace with antigenic drift in HA, and a completely new vaccine is needed in the event of an antigenic shift [1, 2]. Since the manufacture of these vaccines requires 6–9 months from identification of a new strain to distribution, current vaccines cannot be produced rapidly enough to protect against wide-scale mortality and morbidity that generally occurs within the first 3 months after the emergence of a new pandemic strain.

Recent efforts have focused on the development of a new generation of influenza vaccines that could provide broad spectrum, “universal” protection against a wider range of influenza variants including strains with pandemic potential. DNA vaccines possess a number of characteristics that make them particularly well suited for a universal influenza vaccine [3–6]. In the event of a pandemic threat, DNA vaccines offer an important advantage of accelerated vaccine development and production since the DNA vaccine sequences can be obtained directly from the clinical isolate and rapidly constructed and propagated using well-established molecular techniques without the need for cell culture or eggs. DNA vaccines induce both antibody and T cell responses, and both arms of immunity contribute to cross-protection against different influenza variants [7, 8]. Furthermore, many studies have shown that DNA vaccines are highly effective in the induction of CD8+ T cell responses that can play a critical role in rapid clearance of influenza virus, thus limiting pathogenesis [9–12] as well as CD4+ T cell responses that play a key role in maintaining CD8+ T cell memory and providing help for B cells that mediate rapid antibody production [13, 14].

Early studies showed DNA vaccines were poorly immunogenic in humans [15], but recent advances show that this poor performance can be overcome, in part, by improvements in vaccine delivery and co-delivery of adjuvants [16–18]. In contrast to early DNA vaccines administered intramuscularly by needle, DNA administered by electroporation (EP) into the muscle or by particle-mediated epidermal delivery (PMED or gene gun) into the skin more efficiently deliver DNA into cells *in vivo* and have been shown to be capable of inducing strong antibody and/or T cell responses in most subjects and protective levels of immunity in most [4, 19] or 100% [4, 20] of vaccinated humans without the need for an adjuvant or viral vector/protein boosting. PMED influenza DNA vaccine clinical trials showed its possible to induce protective levels of immunity with only a single vaccine dose in humans. Since most of the population has been either vaccinated or previously exposed to influenza, this outcome is likely due to boosting memory responses to other H1 subtypes [4, 21]. However EP and PMED-delivered DNA vaccines are still generally less immunogenic in humans as compared to currently licensed live-attenuated or protein vaccines containing the same antigens [22–24]. Therefore additional strategies, such as co-delivery of adjuvants, are being developed to further increase DNA vaccine potency [6, 15, 25].

Genetic adjuvants are plasmids expressing immune-stimulatory genes that are co-administered with DNA vaccines to increase their immunogenicity [26]. The heat-labile enterotoxin from *E*. *coli* (LT) has been shown to be a powerful adjuvant that can be co-administered as a genetic adjuvant with DNA vaccines [26]. LT and a related adjuvant, cholera toxin (CT), are members of the AB5 class of bacterial toxins [27]. Parenteral or mucosal administration of these adjuvants can be toxic. In contrast, administration of LT to the skin of animal and human subjects is safe, even at high doses (i.e. 500mg) [28]. LT adjuvant activity is mediated in part by direct activation of dendritic cells (DCs) and Langerhans cells (LCs) including LCs and DCs present at cutaneous sites. LT also promotes migration of DCs and LCs to mucosal immune tissue such as Peyers patches [29–32]. The high concentration of LCs, in the skin makes LT particularly attractive for enhancing the mucosal and systemic immunogenicity of vaccines administered via the skin and therefore well-suited to PMED [29, 30, 32]. Consistent with this notion, we previously showed that co-delivery of a plasmid expressing the A and B subunits of LT substantially increases DNA vaccine induction of antibody and T cell responses [26].

A successful universal influenza vaccine will likely need to induce antibody and T-cell responses against multiple conserved antigens. To this end, we designed an LT adjuvanted, multi-antigen (LT-MA) DNA vaccine consisting of four HA genes, the ectodomain of M2 (M2e) and the consensus sequence from the highly conserved influenza A nucleoprotein (NP) [33]. The M2e gene is a 23 amino acid conserved B cell epitope that is poorly immunogenic [34]. To address this, the M2e gene was fused to a gene encoding the highly immunogenic hepatitis core antigen (HBcAg-M2e). This design results in the expression of HBcAg virus like particles carrying M2e epitopes on their surface [35] and is based on studies employing recombinant protein vaccines which have shown that conjugating M2e to HBcAg virus-like particles enhances M2e immunogenicity [36–38]. Similarly, we previously showed that immunizing with hybrid DNA vaccines expressing HBcAg virus like particles carrying HIV and SIV B or T cell epitopes induced stronger responses than DNA vaccines expressing the whole antigen or the epitopes alone [39, 40].

Each vaccine antigen was expressed and co-delivered on separate plasmids to reduce competition between vectors and maximize epitope expression [8]. The four HA genes consisted of 3 previously circulating seasonal H1, H3, and B strains (H1N1-A/New Caledonia/20/99, H3N2-A/Panama/2007/99, B/Jiangsu/10/03) and one avian H5N1 influenza virus that has recently infected humans (H5N1-A/Vietnam/1203/04) [41]. As such, the HA immunogens included in the LT-MA DNA vaccine mimic the composition of a seasonal influenza vaccine. Similar to currently marketed vaccines, HA DNA vaccines induce strong antibody responses against the highly variable HA head that do not contribute to cross-protection. However, HA DNA vaccines have been shown to also induce cross-protective antibody responses against the more conserved stem region of HA [42, 43].

Here, we investigated immunogenicity and protective efficacy of an LT-adjuvanted multi-antigen (LT-MA) universal influenza DNA vaccine consisting of HA, M2, and NP antigens in cynomolgus macaques (*Macaca fascicularis*) that have been used as a preclinical model that closely resembles human immunology and physiology to study vaccine and antiviral efficacy against highly pathogenic human and avian influenza viruses [44–47]. Here, we show that a 3 vaccinations of an LT-adjuvanted DNA vaccine administered 6 weeks apart was able to induce a high titered antibody response. In addition, we show that the adjuvanted DNA vaccine induced mucosal responses and potent, cross-reactive T cell responses that correlate with lower viral loads and less inflammation. These results demonstrate the feasibility of using an adjuvanted universal influenza DNA vaccine for broad protection against drifted and shifted strains of influenza with pandemic potential.

## Results

### LT-adjuvanted multi-antigen DNA vaccine induces strong serum and mucosal antibodies in nonhuman primates

**Fig 1:**
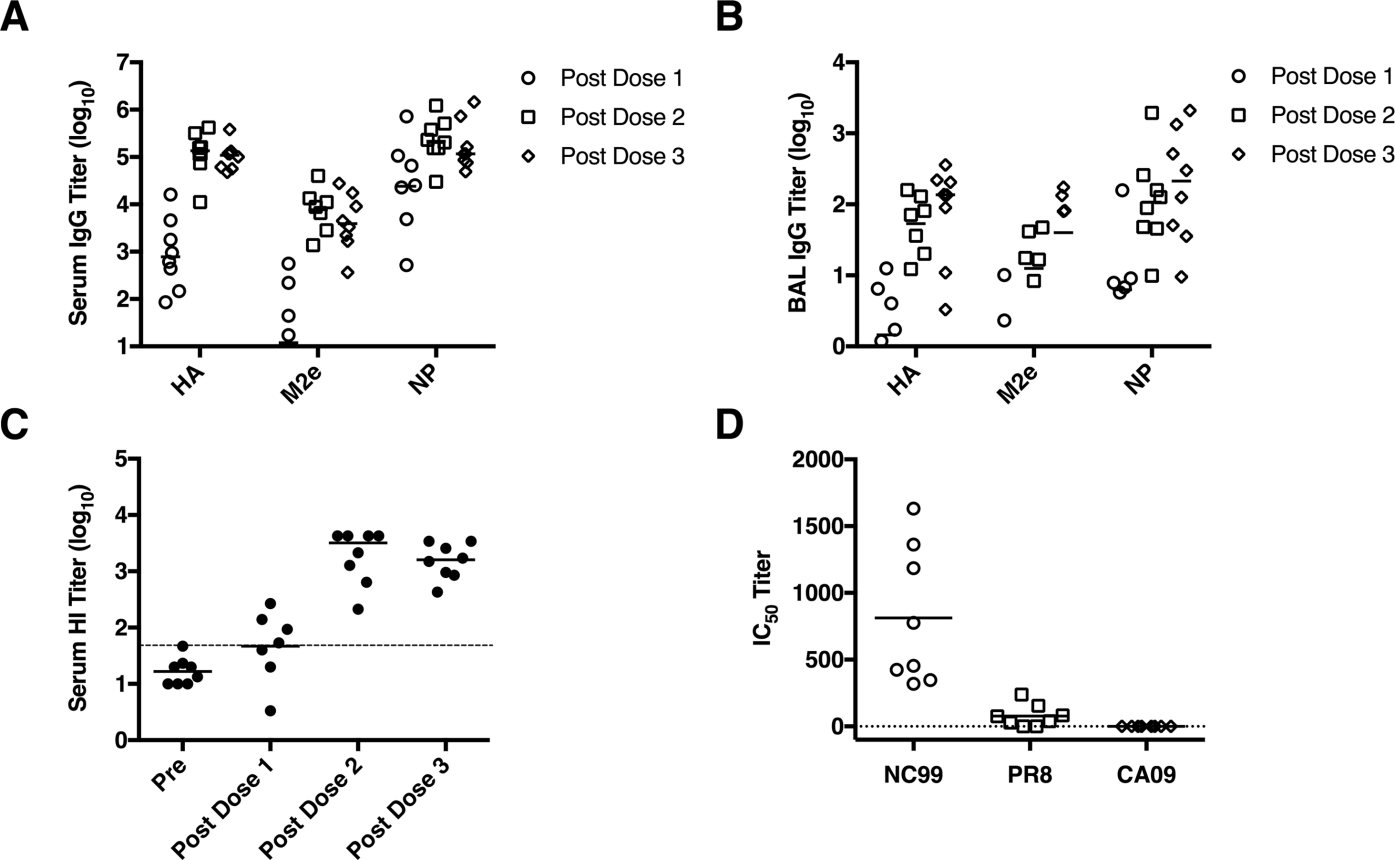
LT-MA DNA vaccine induces systemic and mucosal antibodies in NHP against HA, M2e, and NP. Cynomolgus macaques (n = 8) received 3 immunizations with the LT-MA DNA vaccine at 0, 6, and 12 weeks. Sera was collected from vaccinated macaques at various time-points and analyzed for the presence of IgG by ELISA and hemagglutinin inhibition (HI) assay. A) IgG antibody responses against the vaccine components M2e, NP and the representative HA H1N1-A/New Caledonia/20/99 (NC99) in the serum. Dashed line represents an HI titer of 1:40. B) IgG antibody responses in the bronchioalveolar lavage (BAL) collected at various time-points against the vaccine components M2e, NP and HA (NC99). C) Serum HI titers against NC99 virus. D) IC_50_ titers of vaccinated macaque serum against the matching vaccine strain NC99 and the heterologous unmatched strains PR8 and CA09. Neutralization assays were performed on serum samples taken from vaccinated macaques two weeks after the final immunization.

Studies in mice have shown that influenza DNA vaccines induce strong responses and cross-protection against heterologous challenges [48–52]. However, vaccines that work well in mice are often less effective in humans. The nonhuman primate more closely resembles humans in size, physiology and immune response. We therefore investigated the ability of the LT-MA DNA vaccine to induce antibody and T cell responses in cynomolgus monkeys. To reduce competition between the vectors, each DNA vaccine vector was coated onto separate gold particles with the LT adjuvant at a 10:1 DNA vaccine to adjuvant ratio and then the gold beads were mixed prior to immunization into the skin with PMED. Since PMED results in most cells receiving a single gold bead, this strategy eliminates competition in the expression of multiple plasmids in the same cell. We previously showed this formulation results in expression of each vaccine plasmid in separate cells and induction of immune responses that are comparable to levels induced when immunizing with each plasmid separately [8]. In mice and ferrets, we previously showed that this vaccine induced comparable antibody titers against all 4 HA plasmids included in the vaccine [53]. Macaques received 3 immunizations with the LT-MA DNA vaccine spaced 6 weeks apart. Two weeks after each dose, blood was collected and serum IgG responses were measured by ELISA. After only a single immunization, IgG responses against each component of the vaccine (HA, NP, and M2e) were detected. Antibody responses against each antigen were boosted after the second dose but titers remained unchanged following the third dose in all 8 influenza-naïve animals (**Fig 1A**).

Mucosal IgG responses in the bronchioalveolar lavage (BAL) were measured against M2e, NP and a representative HA antigen H1N1-A/New Caledonia/20/99 (NC99) by ELISA. Mucosal IgG responses were detectable two weeks after the first vaccine dose, increased significantly following the second dose and, in contrast to the serum IgG antibodies, were further boosted following the third dose in 6 of 8 vaccinated macaques (**Fig 1B**). Therefore, the PMED delivered LT-MA DNA vaccine induced HA, M2e and NP-specific mucosal antibody in addition to systemic antibody responses, a result that is consistent with our previous findings showing that PMED induces both mucosal and systemic immune responses in nonhuman primates [8, 54, 55].

HA-specific neutralizing antibody responses were measured by hemagglutinin inhibition (HI) assay which measures neutralizing antibodies against the HA head region. The LT-MA DNA vaccine induced HI antibody titers that greatly exceeded the minimum required for protection in humans (HI titer of 40). HI titers exceeded protective levels in 5/8 (62.5%) macaques after a single dose and in all 8 (100%) macaques after a third dose (**Fig 1C**). Significantly, 6 of these 8 animals developed robust HI titers that exceeded 1,000. These results confirm our previous findings in nonhuman primates showing that 3 doses of PMED DNA vaccination alone, without the need for boosting with recombinant protein or viral vectors, can induce antibody titers that greatly exceed protective levels of immunity [8].

To determine if these strong antibody responses were broadly neutralizing, sera from vaccinated macaques were analyzed for the ability to neutralize homologous and heterologous H1 influenza strains *in vitro*. Consistent with the HI analysis, the sera neutralized the H1N1 virus that is homologous to the H1N1 HA immunogen included in the vaccine (NC99) (**Fig 1D**). However, there was no detectable cross-neutralization of two heterologous H1N1 viruses, A/PR/8/34 (PR8) or A/California/07/09 (CA09), which respectively share 89% and 80% sequence identity to the H1N1 vaccine strain NC99 (**Fig 1D**). These results indicate that the LT-MA DNA vaccine was able to elicit significant levels of neutralizing HA-specific antibodies in the serum of immunized macaques against a matching strain of influenza but not against genetically distinct or shifted strains of influenza.

### LT-MA DNA vaccine induces strong cross-reactive T cell responses in nonhuman primates

**Fig 2:**
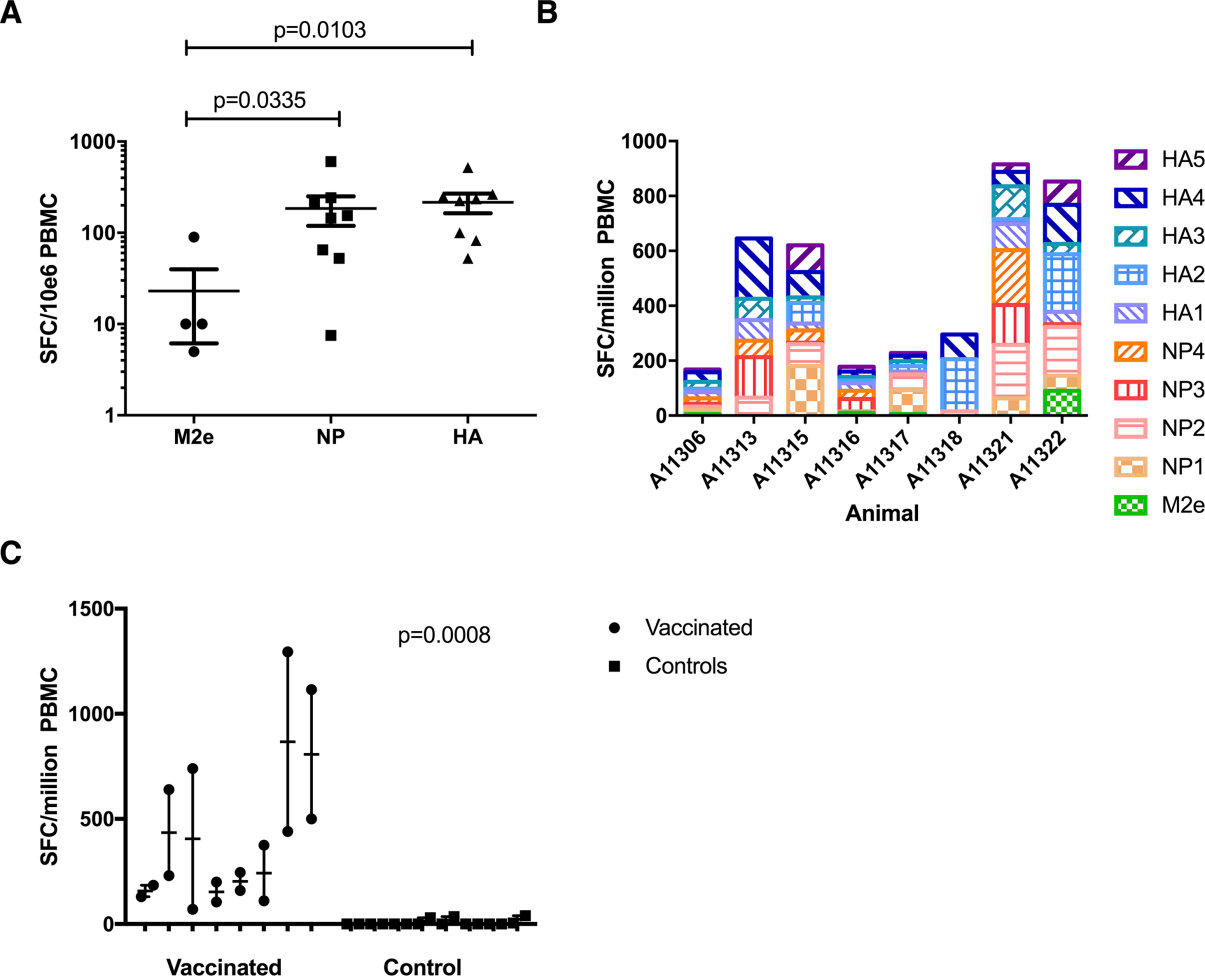
DNA immunization induces influenza-specific T cell responses. A) IFN-**γ** analysis was performed on peripheral blood mononuclear cells. Vaccine-induced responses to specific influenza antigens 2 weeks post third vaccination (week 14). Mean response for each animal is plotted (n = 8/group). Error bars represent SEM. B) Responses of vaccinated animals to influenza peptide pools corresponding to subsets of HA and NP as well as M2e at 2 weeks post third vaccination (week 14). C) Total IFN-**γ** responses against HA, NP, and M2e in vaccinated and control animals at 2 weeks post third vaccination (week 14). Mean of duplicate samples is plotted; error bars represent SEM. Responses after third immunization are significantly elevated over baseline; p = 0.0008 as calculated by Mann-Whitney U test.

To measure induction of cellular immune responses induced in the blood by the vaccine, we performed IFN-**γ** Elispot assays using pools of overlapping peptides comprising the entire amino acid sequence of HA, NP and M2e of the heterologous pandemic swine origin challenge strain, CA09 (H1N1). Modest cross-reactive IFN-**γ** responses were observed 2 weeks after the first vaccine dose, with a robust boost in magnitude after the second vaccine dose. The third vaccine dose did not increase mean IFN-**γ** responses, but responses were clearly boosted in those animals that exhibited low responses after the second dose (data not shown). All 8 animals (100%) exhibited strong cross-reactive IFN-**γ** responses against the heterologous challenge strain (CA09 peptides) following the third dose (**Fig 2A**). The cellular responses observed were largely directed against HA and NP antigens, with only 4 of 8 animals generating M2e-reactive T cells (**Fig 2A and 2B**). However, all 8 vaccinated animals generated broadly reactive cellular responses to multiple HA and NP peptide pools (**Fig 2B**), and IFN-**γ** responses were significantly higher in the vaccinated animals when compared to responses in the unvaccinated, control animals, p = 0.0008 (**Fig 2C**).

### Protection from heterologous influenza challenge

Three weeks post final vaccination (week 15), control and vaccinated macaques (N = 8 macaques per group) were challenged with 10^7.4^ plague forming units (PFU) of the heterologous H1N1 A/California/04/2009 (CA09) virus through a combination of intratracheal, intranasal, ocular and oral routes to mimic the routes humans are naturally infected with influenza [44]. CA09 is a highly virulent Group 1 pandemic influenza strain that shares 80% amino acid sequence identity to the closest related HA immunogen used in the vaccine (H1N1 NC99) so the heterologous challenge with CA09 mimics vaccination against a seasonal strain and subsequent exposure to an unmatched pandemic strain. Infection of cynomolgus macaques with CA09 has been previously shown to result in efficient replication of the virus in the lungs causing mild lung lesions and an influx of inflammatory infiltrates [44].

**Fig 3:**
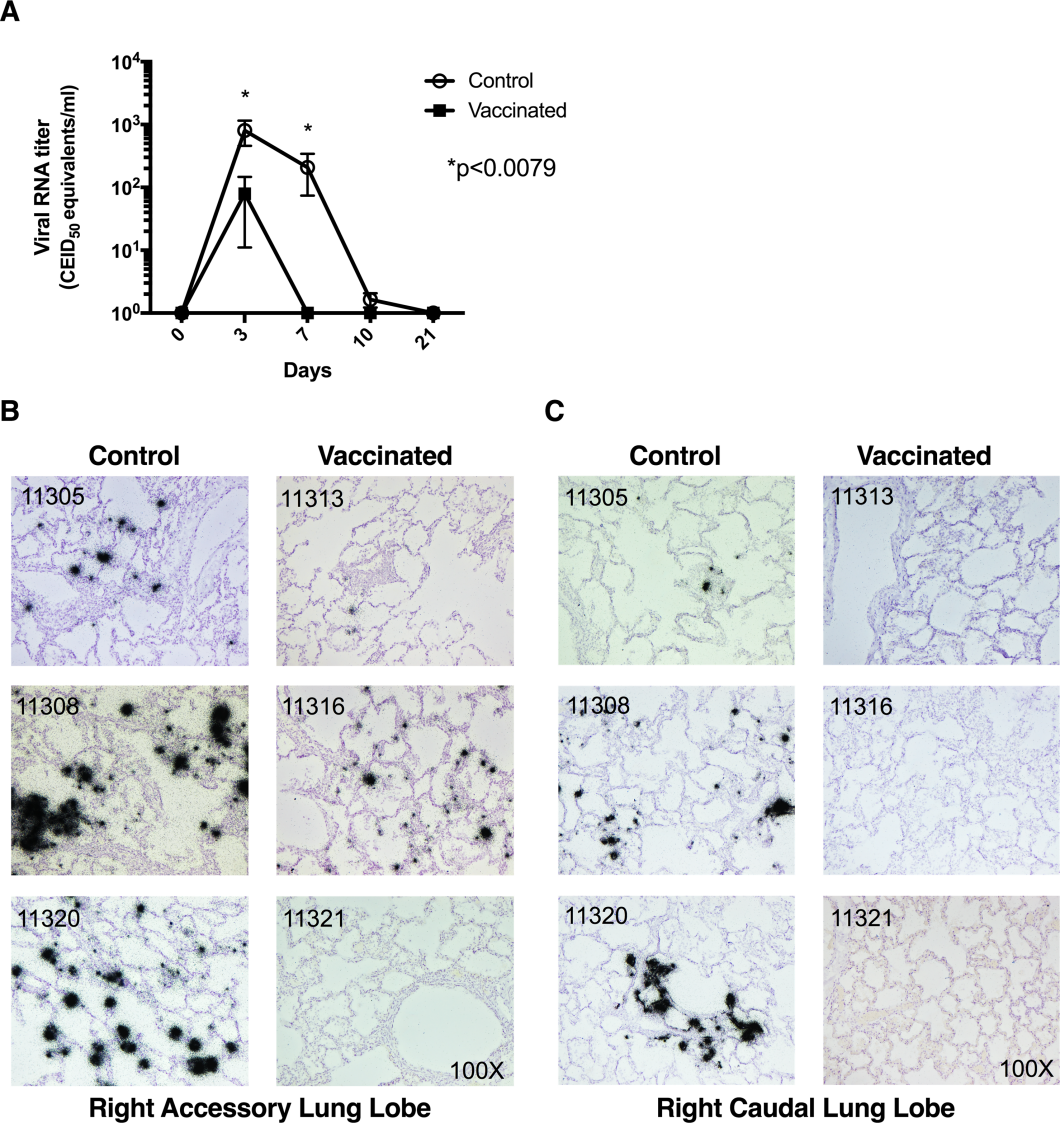
Vaccinated macaques have reduced viral RNA loads and viral replication in nasal washes and lungs. Three weeks post final vaccination (week 15) control and vaccinated macaques are challenged with 10^7.4^ PFU of A/California/04/2009 virus (n = 8/group). A) Bronchioalveolar lavage (BAL) viral RNA titers in control and vaccinated macaques (p<0.0079, Mann-Whitney U test). *In situ* hybridization with influenza virus specific, ^35^S-labeled riboprobes was used to localize challenge virus RNAs in the B) right accessory and C) right caudal lung lobes of control and vaccinated macaques at day 3 post infection (n = 3/group). Viral RNA signals are evident as collections of black silver grains over cells. Animal numbers are noted in the upper portion of each micrograph.

To assess the effects of the vaccine on protection, bronchoalveolar lavage (BAL) samples were collected at various time points post-challenge and analyzed for viral load by semi-quantitative Taqman reverse-transcriptase PCR (RT-PCR). The viral RNA titers in the control group were significantly higher than the vaccinated group at both days 3 and 7 post-challenge (p < 0.0079) (**Fig 3A**). At day 3, three macaques per group were necropsied and the right lung was collected and fixed in neutral buffered formalin to investigate the effects of vaccination on viral replication, tissue damage and inflammation in lung tissues. *In situ* hybridization for influenza virus RNAs was performed on the right accessory (**Fig 3B**), and the right caudal lung lobes (**Fig 3C**). We observed lower viral RNA expression in the lungs of vaccinated macaques when compared to controls for both the right accessory and right caudal lung lobes. These results show that the LT-MA DNA vaccine reduced pulmonary viral loads and accelerated viral clearance demonstrating significant protection from the heterologous challenge. Data shown are representative of the signals observed for each tissue section as whole.

### Vaccinated macaques displayed more rapid response and less inflammation

**Fig 4:**
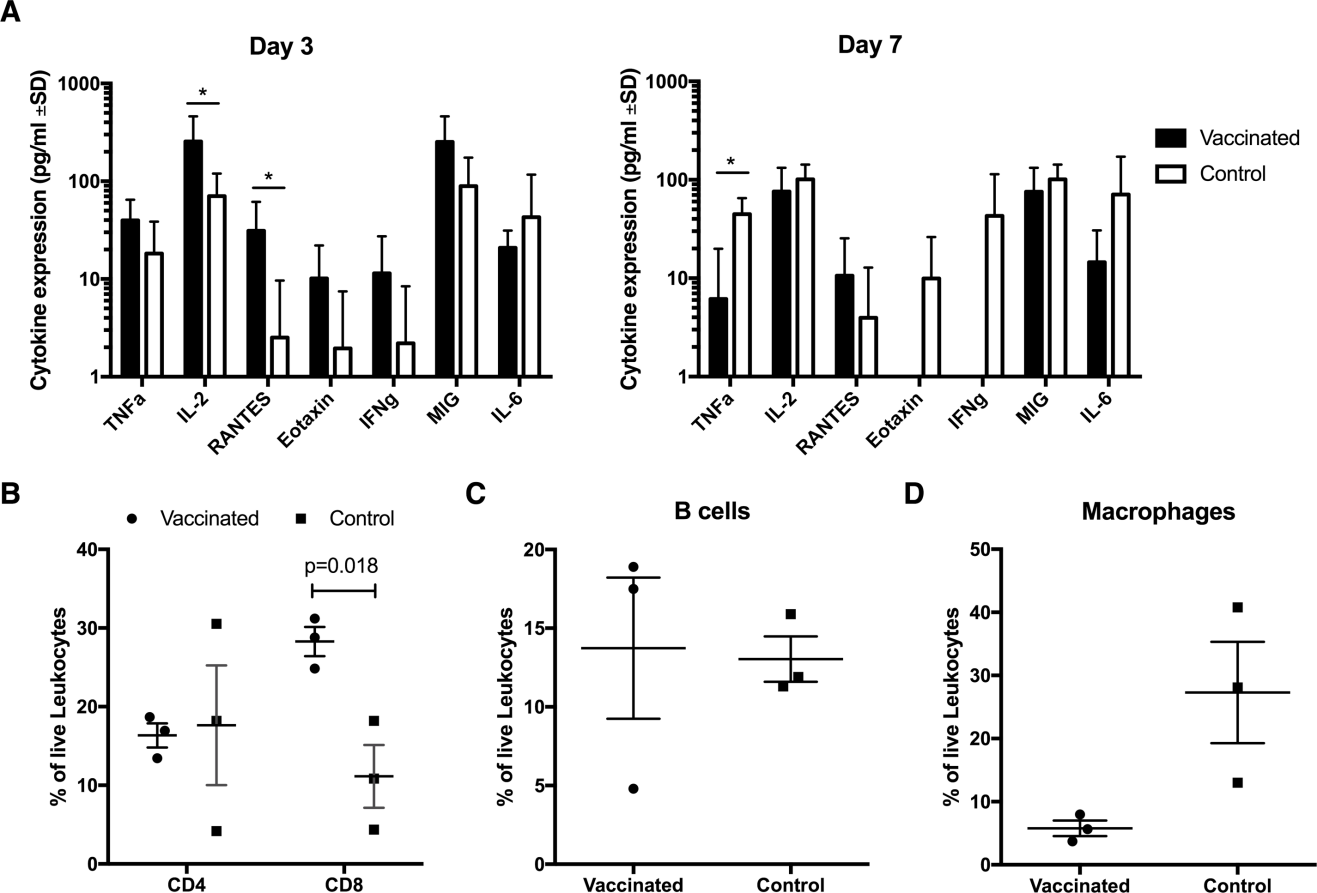
Vaccinated macaques displayed more rapid immune response and less inflammation. A) Inflammatory cytokines were assayed by Bio-Plex using BAL obtained from vaccinated and control macaques on day 3 and day 7 following CA09 challenge (day 3 n = 8 per group, day 7 n = 5 per group). Percentage of B) CD4+ and CD8+ T cells (P = 0.018), C) B cells, and D) macrophages in BAL obtained from vaccinated and control macaques at day 3 post-challenge. *P < 0.05, Mann-Whitney U test. Refer to **S2 Fig** for a gating scheme of representative samples.

Influenza infection results in the induction of inflammatory responses that lead to tissue damage and disease. To determine if the LT-MA DNA vaccine protected from induction of inflammatory responses post-challenge, a multiplex cytokine analysis was performed on BAL samples to assess soluble factors present in the lungs of the macaques at 3, 7, 11, and 21 days after heterologous challenge. As expected, all cytokines influenced by the infection peaked at days 3 or 7 post-infection (**Fig 4A**) and declined to near baseline levels by the later timepoints (data not shown). Importantly, T-cell activating cytokine IL-2 was significantly elevated in the vaccinated animals during acute infection when compared to the controls (p = 0.0264), as was leukocyte chemoattractant RANTES (p = 0.0430) (**Fig 4A**), which is produced by epithelial cells in response to influenza infection in order to recruit leukocytes to the site of infection [56]. On the other hand, Eotaxin, MIG, IL-6 and TNF-**α**, which are pro-inflammatory and chemoattractant responses that are induced by influenza infection, peaked early in the vaccinated group at 3 days post infection but then declined by 7 days post infection. This is in contrast to the control group, where these inflammatory cytokine responses continued to increase or stay elevated between 3–7 days post infection (**Fig 4A**). These results indicate inflammatory responses were more rapidly resolved in the vaccinated animals than in the controls, an outcome that is consistent with the vaccine mediating more rapid viral clearance and reduced lung inflammation.

To confirm these findings, three animals from each treatment group were sacrificed at 3 days post infection to assess lung pathology and immune cell infiltrates (**S2 Table** and **S4 Fig**). Flow cytometric phenotypic analysis of leukocytes isolated from lung tissue revealed significantly more CD8^+^ T lymphocytes present in the lungs of vaccinated macaques as compared to controls (p = 0.018, **Fig 4B**). Statistical significance was difficult to achieve with an n-value of only three animals per group necropsied at this time point. However, the vaccinated animals appeared to have similar levels of CD4^+^ T lymphocyte and CD20^+^ B lymphocyte infiltration, and decreased CD14^+^/CD11b^+^ macrophage recruitment as compared to unvaccinated control animals (**Fig 4B and 4D**). These data support the hypothesis that the LT-MA DNA vaccine induced local mucosal CD8+ T reponses that rapidly recalled in response to challenge, resulting in reduced viral replication and decreased lung inflammation.

### Protection from influenza is mediated by T cell responses

**Fig 5:**
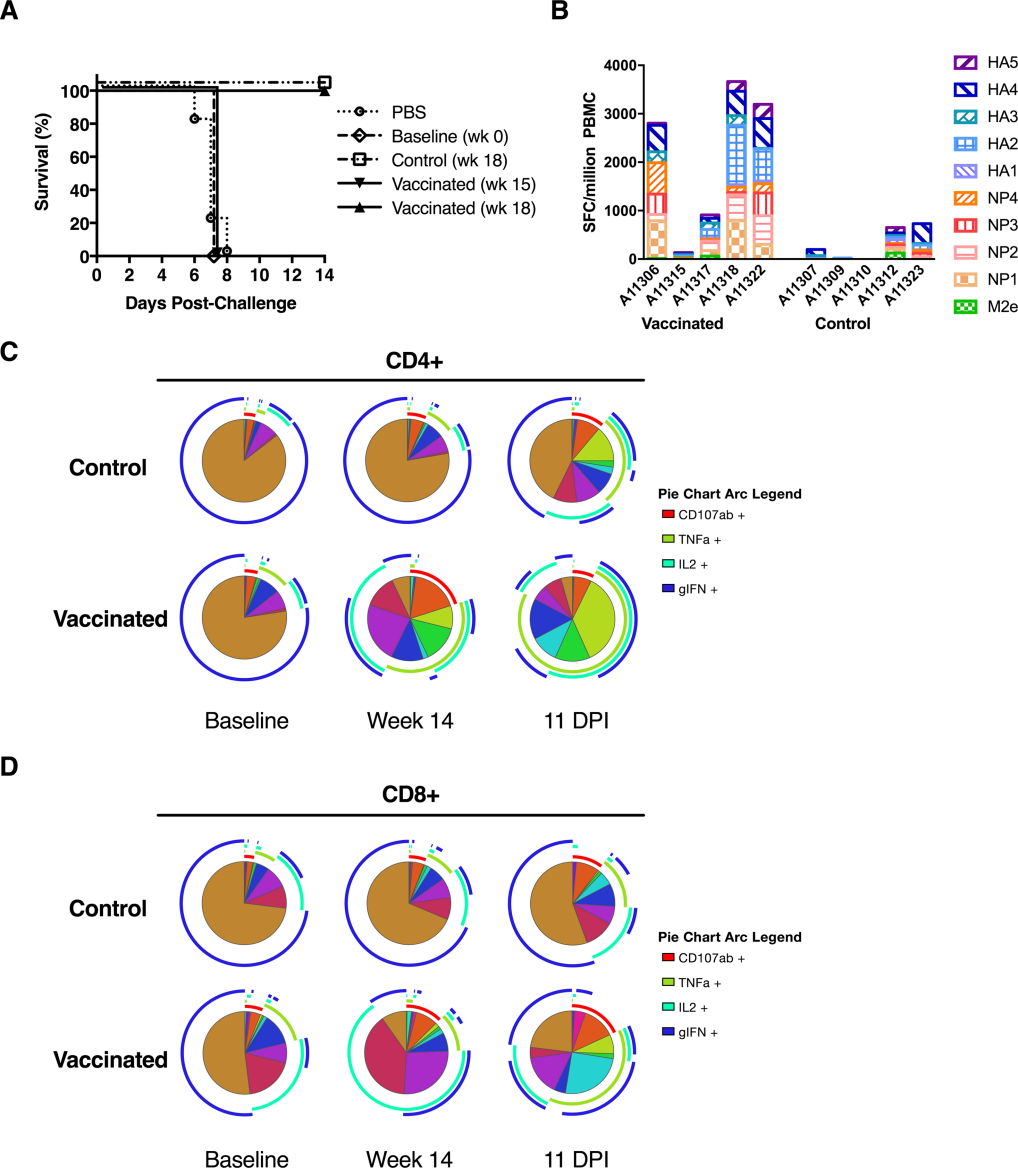
Protection from influenza is mediated by T cell responses and not antibody responses. A) Passive transfer in mice of purified IgG from vaccinated and control macaques followed by influenza challenge with 10 MLD_50_ CA09 virus. Survival of BALB/c mice (n = 10 per group) passively immunized (intraperitoneally) with 25 mg IgG 24 hrs before challenge with a lethal dose of CA09 influenza virus. B) Sum of responses of vaccinated animals to influenza peptide pools corresponding to subsets of HA, NP and M2e at 3 weeks post challenge (week 18) p<0.05. Spice graphs displaying cumulative polyfunctional C) CD4+ and D) CD8+ T cell responses following stimulation with HA, NP, and M2e peptide pools at baseline (week 0), post vaccination (week 14) and 11 days post infection (DPI) (week 16+4 days). The pie charts summarize the cumulative CD4+ and CD8+ T cell responses to the various effector functions as ether mono-or poly-functional responses.

We next investigated immune correlates of protection. The DNA vaccine induced strong cross-reactive T cell responses against the challenge strain, but we were unable to detect broadly neutralizing antibody responses. To further investigate if antibodies contributed to protection, we passively transferred purified IgG from vaccinated and control macaques to naive mice (25 mg/animal) by intraperitoneal (IP) injection 24 hours before lethal challenge with the mouse-adapted CA09 virus. The mouse-adapted CA09 virus leads to rapid weight loss and death in mice within 5–8 days post-infection [57]. IgG was purified from sera collected from vaccinated and control macaques before the first vaccine dose (Baseline, week 0), 3 weeks after final vaccine dose but before challenge (Vaccination, week 15) and 3 weeks after the challenge (Control, week 18) and (Vaccinated, week 18). Naive mice (PBS) received PBS by IP injection 24 hours before CA09 challenge and as expected, lost greater than 25% of their weight and were euthanized within 6–8 days. Similarly, mice that received IgG purified from sera collected from either control or vaccinated animals before the first vaccine dose (week 0) or after the final dose (week 15) succumbed within 7–8 days post-challenge (**Fig 5A**). As expected, IgG purified from sera collected post-challenge (week 18) with CA09 after strong HI titers against the challenge virus arose, protected mice from lethal CA09 challenge (**Fig 5A**). These results confirm that although the LT-MA DNA vaccine induced robust neutralizing antibody responses against the homologous vaccine immunogens, these responses did not contribute to protection against the heterologous virus challenge.

To investigate the contribution of T cell responses to protection, we analyzed the magnitude, function and specificity of T cell responses that developed in response to CA09 three weeks post-challenge in vaccinated and control macaques. Analysis of the magnitude by IFN-**γ** ELISPOT against CA-09-specific HA, NP and M2e peptide pools revealed significantly elevated cumulative IFN-**γ** responses post-challenge in the vaccinated animals when compared to the controls (total responses, p < 0.05) with the greatest proportion of cross-reactive IFN-**γ** responses directed against NP peptide pools (p < 0.02, 2-way ANOVA) (**Fig 5B, orange and red boxes**). These results indicate that the DNA vaccine primed for robust recall T cell responses against the heterologous CA09 challenge strain.

To determine the effects of the DNA vaccine on T cell function, intracellular cytokine staining was used to analyze magnitude, specificity and characterize function of T cell responses in response to stimulation with CA09 peptides. Gating scheme and representative positive and negative controls can be found in **S1 Fig** Vaccination increased the breadth and polyfunctionality of both CD4^+^ and CD8^+^ T cells when compared to the controls (**Fig 5C and 5D**), although the total magnitude of responding cells was unaltered (**S2 Fig**). Specifically, vaccination significantly increased NP-specific CD4^+^ cells expressing TNF-**α**^+^/IL-2^+^/IFN**γ**^+^ (p = 0.008), TNF-**α**^+^/IL-2^+^ (p = 0.048), and IL-2^+^ (p = 0.008) at week 14 (**S3 Fig**). Following challenge, the vaccinated animals demonstrated more polyfunctional effector T cell responses than control animals at 11 days post infection (**Fig 5C and 5D**). Interestingly, both CD8^+^ and CD4^+^ HA-specific cells expressing TNF-**α**^+^/IL-2^+^ (p = 0.048, p = 0.016) were increased in the vaccinated animals compared to controls, as well as NP-specific CD4^+^ cells expressing TNF-**α**^+^/IL-2^+^ (p = 0.032) (**S3 Fig**). These results show that the DNA vaccine induced polyfunctional CD4^+^ and CD8^+^ T cells, expressing IFN-**γ**, TNF, and IL-2 that have been shown to mediate better protection than monofunctional T cells expressing a single cytokine [8].

### Vaccination augments the acute response to viral challenge

**Fig 6:**
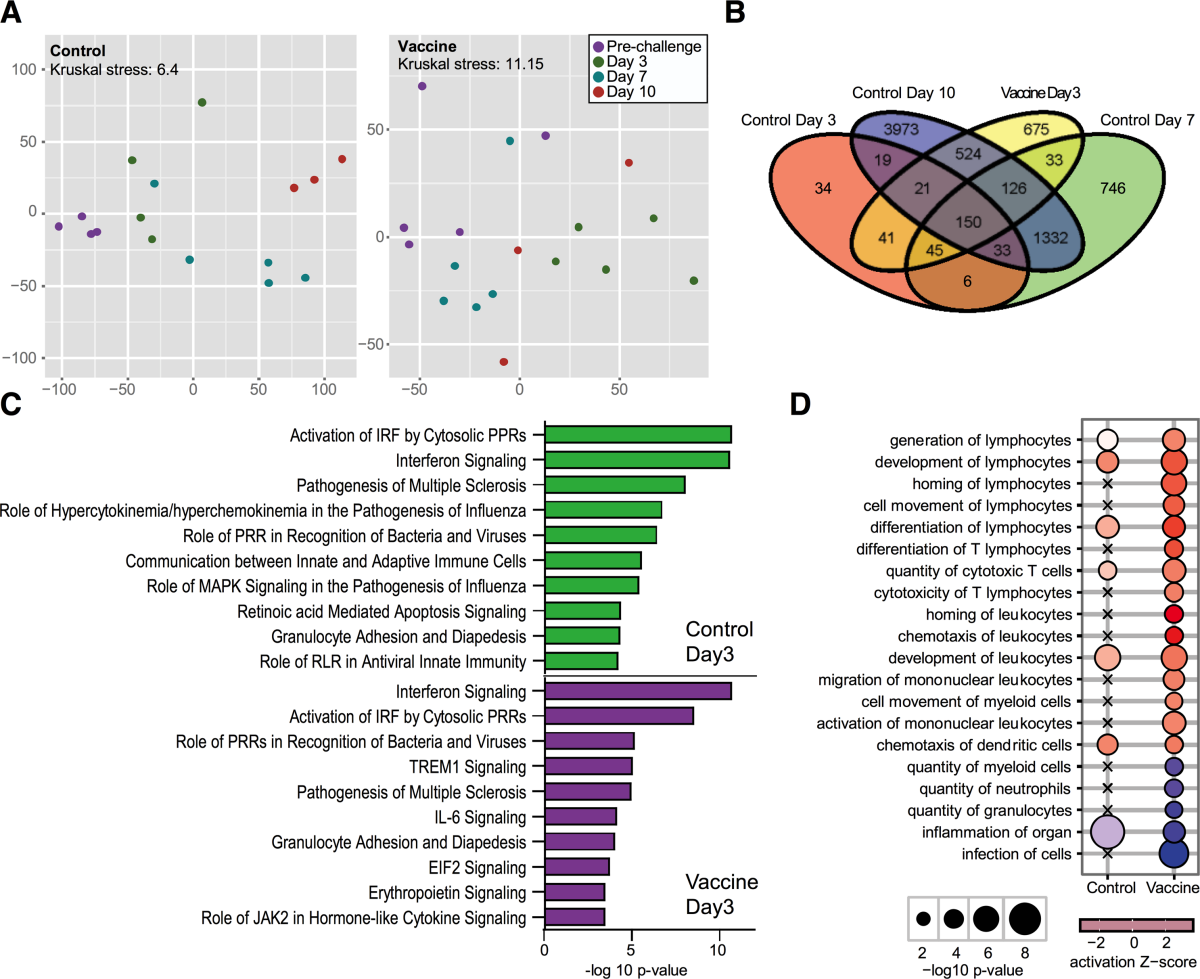
Distinct transcriptional responses following viral challenge in control and vaccinated macaques. A) Expression profiles of the union of 3973 genes found to be differentially expressed were visualized using multidimensional scaling (MDS). The MDS shows similarities between individual profiles. Differential expression cutoff was set to |Fold Change| >1.5 and a q-value < 0.05 calculated using a moderated t-test with Benjamini-Hochberg correction. Each datapoint represents an individual in either the control (left, n = 8) or vaccinated (right, n = 8) group. Distinct color denotes the timepoint at which BAL samples were collected. The Kruskal stress value denotes the quality of the 2D representation of differential gene expression between groups as a fraction of the information lost during the dimensionality reduction procedure. (B) Overlap of DE transcripts in vaccinated and control macaques across time. C) Top 10 canonical pathways found to be significantly enriched at day 3 post challenge in vaccinated and control macaques. Bar graphs represent enrichment scores as determined by the–log10 p-value as calculated by right-tailed Fisher’s exact test. D) Biological functions associated with differentially expressed genes uniquely in vaccinated macaques at day 3 post challenge. Significantly enriched biological functions were those in which |activation z-scores < 2|. Activation z-scores are denoted in by the color of the bubbles. Red indicates upregulation and blue indicates inhibition of biological functions. Bubble diameter represents the–log10 p-value. Crosses signify no observable enrichment of a biological function.

To further probe the impact of PMED vaccination on the host response to viral challenge, we analyzed the BAL-associated genome-wide transcriptional responses at days 3, 7, and 10 post-challenge. Differential gene expression was detected by day 3 following heterologous challenge and continued to increase throughout the course of infection in the control group (**Fig 6A; left**). Host gene expression at 3 days post challenge was more robust in vaccinated macaques than in control macaques (**Fig 6A; right**), which is demonstrated by the degree to which expression of differentially expressed (DE) genes deviate the most from expression prior to challenge. Vaccination led to a rapid change in gene expression, which was not observed until later timepoints (days 7 and 10) in the control group (**Fig 6B**). No DE genes were observed at days 7 and 10 post-challenge in vaccinated macaques, when host transcriptional profiles were found to approximate those observed pre-challenge, a result that is consistent with the observed decreases in viral burden and rapid viral clearance observed in vaccinated macaques relative to control macaques (**Fig 3**).

In both groups, the early response (day 3) to challenge was associated with the induction of pattern recognition receptor and interferon-mediated antiviral responses in both control and vaccinated animals (**Fig 6C**). However, responses in the controls were distinguished by inflammation-associated pathways that are consistent with the pathogenesis of multiple sclerosis (ES = 7.978), hypercytokinemia and hyperchemokinemia in the pathogenesis of influenza (ES = 6.65), and NF-κB signaling (ES = 2.79), and these patterns were enhanced relative to that observed in the vaccinated animals (ES = 4.892, 2.051, and no significant enrichment; respectively). On the other hand, vaccination resulted in greater IL-6 responses that are important for the resolution of influenza-mediated inflammation [58] and the coordination of optimal T cell responses to influenza infection [59]. Lastly, given the observed elevated IFN-**γ** (**Fig 5**) and IL-6 responses with T cell recall responses [60], we further evaluated the impact of vaccination on gene expression associated with the recruitment of leukocytes and lymphocytes into the infected lung and found that the day 3 vaccine transcriptional signatures were consistent with an increase in T lymphocyte recruitment, and cytotoxic T cell stimulation (**Fig 6D**). The vaccinated transcriptional profiles were also predictive of decreased infiltration of myeloid cells, granulocytes, and neutrophils that contribute to inflammation and disease. These data demonstrate that vaccination results in the induction of a robust antiviral response while limiting the recruitment of inflammatory leukocytes [61–64], which is largely coordinated by T cells, thereby limiting viral replication and tissue damage.

### Protection from influenza correlates with NP-specific T cell responses

**Fig 7:**
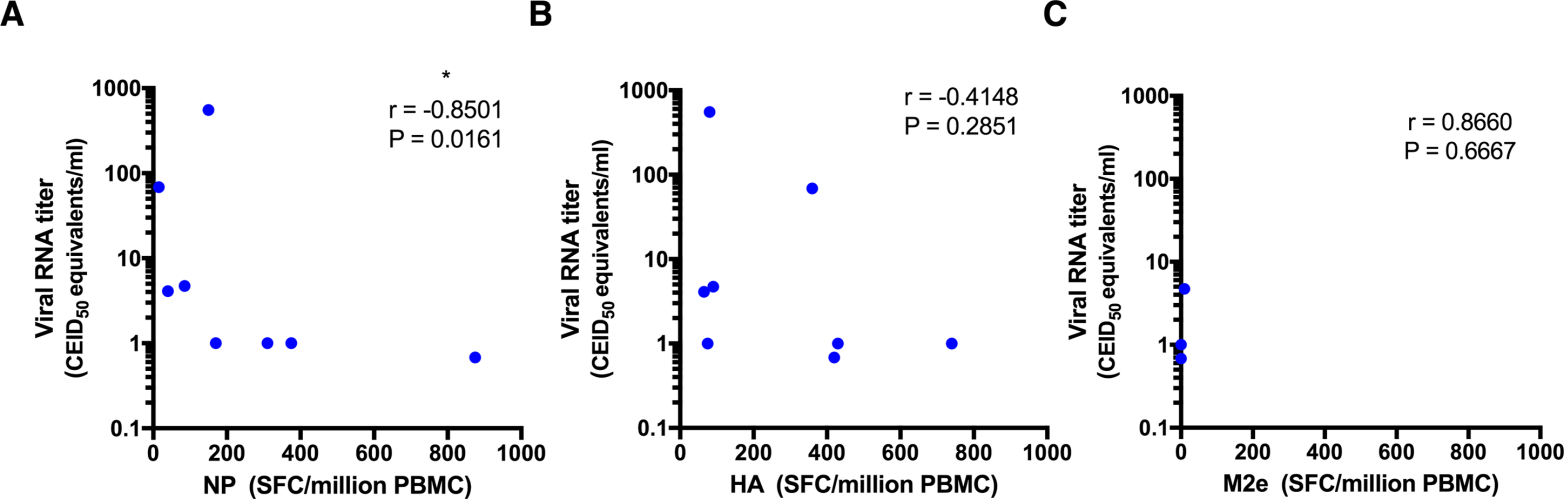
Protection from influenza correlates with NP-specific T cell responses. Correlation between viral RNA titer (CEID_50_ equivalents/ml) on day 3 post-infection and A) NP-specific, B) HA-specific, C) M2e- specific ELISpot IFN-**γ** T cell responses prior to challenge at 2 weeks post third vaccination (week 14) were determined by Spearman-rank correlation tests.

To determine if the magnitude or specificity of pre- or post-challenge T cell responses correlate with protection, we compared T cell responses measured by IFN-**γ** ELISPOT after the final DNA vaccine dose at 3 weeks post-challenge to viral loads measured 3 days post-infection. The results in **Fig 7A** show a significant inverse correlation between the viral loads and the NP-specific IFN-**γ** T cell responses measured only after the final DNA vaccination (p = 0.0161, r = -0.8501) but not 3 weeks post-challenge (P = 0.2134, r = 0.-6727) (data not shown). In contrast there was no statistically significant correlation between the viral load and the magnitude of HA-specific or M2e-specific IFN-**γ** T cell responses measured post-vaccination or 3 weeks post-challenge (**Fig 7A and 7C**). We also compared antibody responses measured by ELISA or HIA, monofunctional and polyfunctional T cell responses, cytokine responses measured at week 15 by Bio-Plex, and overall frequency of CD4+, CD8+, B cell and macrophage present in the lung at week 15 by immunophenotyping and observed no statistically significant correlations (not shown). Taken together, these results indicate that vaccine-induced NP-specific IFN-**γ** T cell responses provided a primary mechanism of protection in this study.

## Discussion

Neutralizing antibodies mediate effective protection against influenza, and it is generally expected that a successful universal influenza vaccine that protects against both seasonal antigenic drift and emerging influenza pandemics will likely need to induce both broadly neutralizing antibody to blunt initial viral replication and cross-reactive CD8+ T cell responses to help clear the cells that become infected. Here, we show in a preclinical nonhuman primate model that an adjuvanted DNA vaccine expressing multiple antigens (HA, M2e and NP) induced very high antibody titers and robust cross-reactive T cell responses but failed to induce broadly neutralizing antibody. Nevertheless, this vaccine was still able to exert significant cross-strain protection, including both early blunting of viremia and accelerated viral clearance, that; correlated with T cell responses, against a challenge with a heterologous pandemic H1N1 strain that shares 85% identity with the most similar H1N1 HA antigen included in the vaccine.

DNA vaccines generally induce modest or low antibody responses when compared to other vaccine modalities [3]. To address this deficiency, DNA vaccines have been combined with other types of vaccines, such as live viral vectors or recombinant proteins in a prime-boost regimen that results in a synergistic induction of robust antibody that is higher than levels induced by either vaccine alone [42, 43, 65]. DNA vaccine priming also appears to expand the antibody epitope repertoire, increase affinity maturation and direct development of T cells with effector memory phenotypes [66–69]. The generation of broadly neutralizing antibodies by heterologous prime-boost regimens was previously found to be dependent on the DNA priming, and the hypothesis emerging from these studies is that the DNA vaccine increases the number and diversity of the CD4+ and B cell clones [42, 70] while the protein or viral vector boosting is needed to expand these clones to get to a threshold titer that can afford cross-strain protection. Here, using a potent genetic adjuvant and highly efficient DNA vaccine delivery technology, we achieved robust induction of homologous HA-specific antibody responses against the strains included in the vaccine with DNA alone (titers > 1:1000). These titers substantially exceeded protective levels in humans (1:40) and were comparable in magnitude to levels previously reported to be induced by DNA priming followed by heterologous protein or viral vectored vaccines [42, 71]. However, despite achieving very high titers, DNA vaccination alone did not induce broadly neutralizing antibodies,. This contrasts to previous work showing that an influenza DNA vaccine prime followed by a boost with either a replication defective Ad5 vector encoding the same HA or the seasonal influenza vaccine induced broadly neutralizing antibodies targeting conserved HA stalk in mice, ferrets and non-human primates [42]. Our study shows it’s possible for a DNA vaccine alone to achieve high titered antibody responses on par with heterologous prime-boost regimens [72, 73] but these responses were not broadly neutralizing. The mechansims underlying the synergy between DNA vaccines and protein or viral vectored vaccines in heterologous prime-boost regimens are still unknown. Our results suggest that while DNA vaccines can prime for broadly specific antibody and T cell repertoire, the diverse antigen presentation pathway provided by a heterologous prime-boost regimen may be key to expand these responses. Recent studies demonstrate the feasibility of inducing even broader neutralizing antibody by using immunogens that present only the HA stalk or by sequential immunization with chimeric vaccines consisting of divergent heads but the same stalk. Such immunogens could be expressed in our DNA vaccine platform resulting in a potent universal influenza vaccine capable of inducing high titered broadly neutralizing antibody and cross-protective T cell responses in a single vaccine modality.

Three doses were employed in this study but we previously showed that PMED delivery of a single dose of an HA DNA vaccine induced protective levels of antibody against a matching strain in humans [4, 5], likely due to the HA DNA vaccine boosting responses that were primed by previous exposures to influenza or prior vaccinations. For HA DNA vaccines designed to match circulating strains, rapid manufacture would provide an advantage over existing vaccines that require 6–9 months to produce. Here, our goal was to determine if incorporating conserved viral antigens into a representative seasonal HA DNA vaccine could provide broader protection against unmatched strains. Since these antigens are conserved, they would not be constrained to the same short manufacturing or distribution window that the more variable HA antigens are and could be manufactured and administered to the population prior to the flu season or concurrently with the seasonal vaccine as we performed in this study. We employed a multi-dose regimen because multiple doses over a longer window of time induce more durable immunity which is the goal of a universal influenza vaccine. Our results show that the PMED LT-MA DNA vaccine alone was able to induce robust T cells that were broad in specificity with the ability to cross-react to a variety of sequences and antigens in a heterologous influenza strain. This result is consistent with our previous studies showing an adjuvanted HA DNA vaccine administered by PMED induced broadly specific T cell responses against multiple sequences in HA and administration of a therapeutic PMED DNA vaccine induced broadly specific T cell responses in SIV-infected macaques that correlated with improved viral control [26, 74].

Significant T cell responses were induced after only 1–2 doses in all animals, suggesting that cross-protective T cell immunity by this vaccine could potentially be achieved with fewer doses, especially in individuals that may be primed for these responses by previous exposure to influenza. Additional studies will be needed to determine the minimum doses needed to induce cross-protection by this vaccine. Studies in mice and humans have shown that in the absence of effective antibody-mediated protection, T cell responses do not prevent or blunt the initial infection but can mediate accelerated clearance of an established infection and reduce inflammation [75]. Consistent with these observations, a previous study in nonhuman primates showed that a DNA vaccine that induced strong systemic T cell responses but not neutralizing antibody afforded mild protection against a heterosubtypic influenza challenge in that there was no reduction in peak viral shedding at days 3–5 but enhanced viral clearance later in the course of the infection [76]. In contrast, the adjuvanted DNA vaccine in our study not only enhanced clearance of the infection but provided significant early blunting of acute viral replication within the first 3 days post-infection that is more similar to the expected effects of antibody. Concurrent with this effect, we also observed rapid recall T cell responses post-challenge, an early influx of CD8+ T cells into the lung and early induction of the pro-inflammatory cytokines in vaccinated macaques compared to controls that correlated with dampened recruitment of inflammatory leukocytes, reduced viral replication and protection from lung tissue damage. These results show the DNA vaccine was able to induce a robust early antiviral response that blunted acute viremia in the lung very early in infection. Since we detected no neutralizing antibody against the challenge strain but strong cross-reactive T cell responses and rapid influx of CD8+ T cells to the lung post-challenge, these results suggest cross-reactive local effector T cell responses induced by the vaccine likely blunted the acute viremia at the mucosal site of viral exposure. Consistent with this possibility, a previous study in nonhuman primates showed that mucosal exposure to subpathogenic doses of influenza virus induces lung resident influenza-specific T cells that mediated a similar blunting of acute viremia in the lung when animals were subsequently challenged with a high dose heterosubtypic pandemic strain of influenza [75].

Taken together, our results demonstrate that an LT-MA DNA vaccine induced mucosal and systemic antibody responses against the vaccine strains and robust cross-reactive T cell responses that even in the complete absence of broadly neutralizing antibody can still mediate robust heterologous protection from a highly divergent strain of influenza. These results make a strong case for generating a universal influenza vaccine that induces cross-reactive T cell responses to enhance existing antibody-based modalities. A universal influenza DNA vaccine that induces robust cross-reactive T cell responses against influenza antigens, in addition to strong antibody responses against homologous strains, could provide an important alternative to existing influenza vaccines by providing antibody-mediated protection against seasonal strains of influenza and at the same time, inducing T cell responses that could provide added protection against emerging or unexpected pandemics.

## Materials and methods

### Research animals and ethics statement

All animal experiments used in this study were approved by the University of Washington Institutional Animal Care and Use Committee (protocol# 4266–02), and in were in compliance with the U.S. Department of Health and Human Services Guide for the Care and Use of Laboratory Animals and Animal Welfare Act. This study used n = 16 male cynomolgus macaques (*Macaca fascicularis*) between the ages of 4 and 6 (average 5). All animals were pre-screened for cross-reactive antibody to influenza antigens prior to enrollment in the study. Animals were singly housed in comfortable, clean, adequate-sized cage. Cages, racks, and accessories were sanitized in mechanical cage washers at least once every two weeks and waste pans are cleaned daily. Temperature in animal quarters was maintained at 72–82°F. Animals were fed a commercial monkey chow, supplemented daily with fruits and vegetables and drinking water was available at all times provided by automatic watering devices. Throughout the study, animals were checked twice daily by the veterinary technicians to evaluate their physical and clinical condition. Environmental enrichment activities included grooming contact, perches, toys, foraging experiences and access to additional environment enrichment devices such as paint rollers, grooming devices, foraging devices, activity panels and mirrors. All procedures were performed either under ketamine sedation (10 mg/kg) or Telazol 2.5-10mg/kg to minimize pain. Euthanasia prior to necropsy was performed by administration Euthanol® (Virbac Corp., Houston, TX) while the animal was under deep anesthesia in accordance with guidelines established by the 2007 American Veterinary Medical Association Guidelines on Euthanasia which is consistent with the guidelines described in the Weatherall Report on The Use of Nonhuman Primates in Research. None of the animals became severely ill during the course of the study and none required euthanasia prior to their experimental endpoint.

### Plasmid construction and vaccine formulation

Codon optimized coding sequences for the hemagglutinin antigens, and a NP consensus sequence determined by alignment of the NP protein sequences of H1N1-A/New Caledonia/20/99, H3N2-A/Panama/2007/99, and H5N1-A/Vietnam/1203/04 were designed and constructed by GeneArt. The coding sequences were inserted into the Nhe1 and Bgl2 sites of PJV7563 [8], an empty DNA vaccine expression plasmid. HBcAg-M2e was constructed by inserting oligonucleotides that code for M2e into the Bsp120I site of plasmid PJV7063 [40], resulting in a plasmid that expresses a fusion of M2e in the immunodominant loop of HBcAg. The construction of the LT plasmid is described elsewhere [77]. Plasmid DNA was precipitated onto 1–3 **μ**M gold particles as previously described [22] at a rate of 1.8 **μ**g of each antigen plasmid and 0.2 **μ**g LT adjuvant plasmid (10:1 ratio of DNA vaccine to adjuvant) per 1.0mg of gold. This study was investigated in 2 cohorts (Vaccinated and Control) of cynomolgus macaques with N = 8 animals per cohort. Each cohort consisted of 4 animals from each of the 2 treatment groups (total of N = 8 animals/group). Animals were stratified to provide comparable distribution of weight and age into each group. Animals (Vaccinated group n = 8) were sedated, the leg and abdominal fur was clipped, and DNA-coated gold particles were accelerated into the skin of the abdominal and inguinal regions using a gene gun PMED device. DNA-coated gold particles were delivered at a helium pressure of 45 bar psi. Each actuation resulted in the delivery of 2 **μ**g of DNA into the epidermis. A single dose consisted of 16 actuations for a sum of 32 **μ**g. Each dose was administered 6 weeks apart.

### Producton of H1N1 Influenza A Virus

Sub-confluent MDCK cell monolayers in T75 flasks were washed and inoculated with 2ml serum-free MEM containing 0.5 multiplicity of infection (MOI) A/California/07/2009 (CA09) H1N1 Influenza A Virus obtained from J.T. Weinfurter (Wisconsin Primate Center, UW Madison) for 1 hour at 37°C/5% CO2 [75]. The virus and cell inoculum was rocked every 15 minutes to avoid drying of the monolayer and then 18 ml additional medium was added to each flask and the culture medium was harvested at 36 hours post-infection. Cell debris was pelleted at 200rpm for 10min at 4°C, and then filter-sterilized through a 0.22micron filter. Virus stock was frozen at -80°C in 1ml aliquots, and titered by plaque assay on MDCK cells.

### Experimental challenge of cynomolgus macaques with influenza virus

Eight macaques per group (Vaccinated and Control) were intramuscularly anaesthetized with ketamine (10 mg/kg) and inoculated with a suspension containing 10^6.5^ p.f.u. ml^-1^ of CA09 virus through a combination of intratracheal (4.5 ml), intranasal (0.5 ml per nostril), ocular (0.1 ml per eye) and oral (1 ml) routes (resulting in a total infectious dose of 10^7.4^ PFU). Macaques were monitored daily for clinical signs of disease including, sneezing, nasal discharge, weight loss and activity level. On days 0, 3, 7, 10 and 21 after infection, nasal and tracheal swabs and bronchial brush samples were collected. On day 3 after infection, 3 macaques per group were necropsied for virological and pathological examinations.

### Enzyme-linked immunosorbent assay (ELISA)

HA-specific IgG antibody levels in mouse and macaque serum and bronchioalveolar lavage (BAL) were assessed by enzyme-linked immunosorbent assay (ELISA). Maxisorp plates (Thermo Scientific-Nunc) were coated with 100 ng/well of recombinant A/New Caledonia/20/99 (Protein Sciences), M2e (Selleck Chemical), or NP in PBS overnight at 4°C. NP protein was made in *E*. *coli* using a his-tagged protein and nickel resin for purification. Plates were blocked with 5% nonfat milk powder in PBS for 1 h at room temperature, and then washed three times with wash buffer (PBS-T; phosphate-buffered saline containing 0.05% Tween 20). Three-fold serial dilutions of samples were added to the wells starting at a dilution of 1:500, and plates were incubated for 1 hr at room temperature. Following three washes with PBS-T, plates were incubated with horseradish-peroxidase conjugated goat anti-macaque IgG (1/5,000 dilution) secondary antibodies (Nordic Immunological Laboratories) for 1 hr at room temperature. After five washes with PBS-T, TMB substrate (KPL) was added to the wells for 30 min at room temperature. Color development was stopped by the addition of TMB Stop solution (KPL), and the plates were read at 450 nm. Antibody endpoint titers were calculated by nonlinear regression analysis of curves using GraphPad Prism and were determined to be two standard deviations above the baseline.

### Hemagglutinin inhibition assay

RDE (Accurate Chemical & Scientific Corp.) treated macaque sera were analyzed for the presence of influenza A/New Caledonia/20/99 specific antibody using a hemagglutination inhibition (HI) assay as described [5]. Briefly, two-fold serial dilutions of RDE-treated sera were incubated with 4 hemagglutination units of influenza A/New Caledonia/20/99 virus. After 30 min at room temperature, 50 μl of 0.5% turkey RBCs (Lampire Biological Laboratories) suspended in PBS was added to each well and incubated for an additional 30 minutes. Serum HAI titers are reported from the average of duplicate tests as the reciprocal dilution of serum found to inhibit hemagglutination.

### Neutralization assay

Neutralization assays were performed using the PB1flank-eGFP viruses as previously described [78]. Briefly, macaque serum was heat inactivated at 56°C for 1 hr, then five-fold diluted in a 96-well plate, and virus was added at an MOI that ranged from 0.1 to 0.8 for the different viruses. Plates were incubated at 37°C for 1 hr to allow antibody binding, and then 4 × 10^4^ MDCK-SIAT1-CMV-PB1 cells were added per well. After an 18-hr incubation, GFP fluorescence intensity was measured using an excitation wavelength of 485 nm and an emission wavelength of 515 nm. Values are reported as percent infectivity remaining averaged over triplicate measurements.

### Passive transfer studies

Sera from vaccinated and control macaques were collected at week 0 (Baseline, vaccinated and control macaques before vaccination and challenge, n = 16), week 15 (Pre-Vaccinated, sera from vaccinated macaques pre-challenge only, n = 8), week 18 (Post-Vaccinated, sera from vaccinated macaques post-challenge, n = 5) and week 18 (Post-Controls, sera from control macaques post-challenge, n = 5). IgG from immune sera was purified with protein G (Life Technologies) using the manufacturer's protocol. Female BALB/c mice (n = 10/group) received saline (Ctr) or 25 mg/animal of either Baseline, Pre-Vaccinated, Post-Vaccinated or Post-Control IgG via an intraperitoneal route. 24 hrs post passive transfer mice were challenge with a lethal dose (10 times the 50% mouse lethal dose or 10 MLD_50_) of the mouse adapted H1N1 A/California/04/2009 (CA09) virus [57]. The mice were monitored daily for weight loss and survival until 14 days post-infection. Animals that lost more than 30% of their initial body weight were euthanized by carbon dioxide in accordance with our animal protocols.

### Isolation of mononuclear cells from blood

Peripheral blood mononuclear cells (PBMC) were isolated by Ficoll density centrifugation. Erythrocytes were removed using ACK lysis buffer (BioWhittaker) and remaining cells washed with RPMI-1640 supplemented with 10% fetal bovine serum.

### ELISPOT

PBMC’s were stimulated in duplicate at 2e5 and 1e5 cells per well with 10 individual peptide pools (1**μ**g/mL each peptide) spanning the full amino acid sequences of HA, NP, and M2e. HA and NP peptides were 15-mers overlapping by 11 amino acids (BEI Resources), M2e peptides were 10-mers overlapping by 9 (Mimotopes). Each pool contained 14–27 individual peptides and sequentially spanned the entire protein. Concanavalin A (Sigma) was used as a positive stimulation control (5**μ**g/mL). DMSO served as a negative solvent control. Antigen-specific T cells secreting IFN-**γ** were detecting using paired anti-macaque IFN-**γ** monoclonal antibodies (U-cytech-BV) as previously described. Spot forming cells (SFC) were enumerated using an Immunospot Analyzer with CTL Immunospot Profession Software (Cellular Technology Ltd.). Results are expressed as the mean number of SFC in replicate wells containing antigenic peptide, subtracting the number of spots from DMSO control wells from the same animal.

### Intracellular Cytokine Staining (ICS)

Cryopreserved PBMC’s were thawed and rested for four hours and 1.2 million cells were stimulated with DMSO, Staphylococcal enterotoxin B (SEB), or peptides (1 **μ**g/mL) each for 1 hour with CD107a/b FITC (H4A3/H4B4, BD) in serum free fully defined AIM V® (ThermoFirsher®) media prior to adding 1 mg/mL of Brefeldin A. Cells were stimulated in the presence of Brefeldin A (Sigma-Aldrich®) overnight at 37C and 5% CO_2_. Viable cells were stained using a LIVE/DEAD® Yellow (ThermoFisher®) amine dye then surface stained with CD3 V500 (Sp34-2, BD Biosciences), CD4 PerCPCy5.5 (L200, BD Biosciences), CD8 APC-Cy7 (RPA-T8, BD Biosciences), CD28 PE-CF594 (CD28.2, BD Biosciences), CD95 BV421 (Dx2, BD). Cells were permeablizied with Cytofix/Cytoperm (BD Biosciences) and stained with an intracellular antibody cocktail containing IFN-**γ** BV650 (4S.B3, Biolegend), TNF-**α** PE-Cy7 (Mab11, BD Biosciences), IL-2 PE (MQ1-17H12, BD Biosciences), Ki67 AF700 (B56, BD Biosciences) in Perm/Wash^TM^ Buffer (BD Bioscinces). See **S1 Table** for antibody concentrations. Cells were then washed with Perm/Wash^TM^ Buffer (BD Bioscinces) then fixed with 1% paraformaldehyde (Sigma) and collected on an LSR II (BD Bioscinces). Flow cytometry was analyzed in FlowJo (Version 9.7.6, Treestar Inc., Ashland, Oregon). See **S1 Fig** for gating and representative positive and negative controls. Graphs represent background (DMSO) subtracted stimulations. A minimum of 100,000 viable T cells were collected for each stimulation.

### Immunophenotyping

Cryopreserved BAL were thawed, seeded 1.2 million cells per well, and rested overnight at 37C and 5% CO_2_. Viable cells were stained using a LIVE/DEAD® Yellow (ThermoFisher®) amine dye then surface stained with CD3-PECF594 (Sp34-2), CD4 eluor605NC (OKT4), CD8-V500 (RPA-T8), CD20-PECy7 (2H7), HLA-DR-BV711 (G46-6), CD14-BV785 (M5E2), CD11b-APC-Cy7 (M1/70). Cells were permeablizied with Cytofix/Cytoperm (BD Biosciences) and stained with an intracellular with Ki67 AF700 (B56) in Perm/Wash^TM^ Buffer (BD Bioscinces). See **S2 Table** for antibody concentrations. Cells were then washed with Perm/Wash^TM^ Buffer (BD Bioscinces) then fixed with 1% paraformaldehyde (Sigma) and collected on an LSR II (BD Bioscinces). Flow cytometry was analyzed in FlowJo (Version 9.7.6, Treestar Inc., Ashland, Oregon). See **S2 Fig** for gating scheme.

### Viral titers by real-time RT-PCR

Viral loads were measured in bronchoalveolar lavage (BAL) samples by semi-quantitative real-time RT-PCR using the CDC protocol for swine influenza A (H1N1) (kit obtained from BEI Repository NR-15577) [79]. Briefly, RNA was extracted from BAL samples using a modified version of the Invitrogen PureLink Viral RNA/DNA kit (cat. # 12280–050). Semi-quantitative RT-PCR was performed using Invitrogen SuperScript III Platinum One-Step Quantitative Kit and Swine Influenza H1 (swH1) primers and probe from the BEI kit. A standard curve was generated by extracting RNA from 10-fold dilutions of CA09 virus of known chicken egg infectious dose 50 (CEID_50_). The relative CEID_50_ of unknown NHP samples were back calculated by comparison with CT values from this standard curve and are expressed as CEID_50_ equivalents/ml. No template and positive (a sample from a prior experiment) template controls were included in each PCR run.

### *In situ* hybridization for influenza virus RNAs

In situ hybridization (ISH) was performed using ^35^S-labeled riboprobes to detect influenza virus RNAs in sections of formalin-fixed, paraffin-embedded lung tissues as described (Fallert 2002 J Virol Methods; Reinhart 2002 Blood). Following stringent hybridization and washing, autoradiographic exposure times were 14 days.

The following primers were used to obtain influenza virus partial cDNAs from viral segments 4 and 5: H1N1 Cal 2009 seg4 HA forward, 5’-TAA GAA GGC AAT ACT AGT AGT TCT GC-3’; H1N1 Cal 2009 seg4 HA reverse, 5’-TGC TAT TTC CGG CTT GAA CT-3’; H1N1 Cal 2009 seg5 NP forward, 5'-TAA GGC GTC TCA AGG CAC CAA ACG A-3'; and H1N1 Cal 2009 seg5 NP reverse, 5’-CAT TTT CAC CCC TCC AGA AA-3’. RT-PCR was performed using total RNA prepared from infected cells as template for cDNA synthesis followed by PCR. The resulting PCR products were agarose gel purified, ligated to pGEM-T vector (Promega) and DNA sequenced. The resulting cDNA-containing plasmids were linearized by restriction digestion and used as template for in vitro transcription to generate sense and anti-sense riboprobes.

### Bio-Plex analysis of cytokine production in BAL

The concentrations of cytokines in the lung were measured by analyzing BAL on days 0, 3, 7, 11 and 21 days post-infection. The levels of interleukin (IL)-2, IL-6, interferon (IFN)-**γ**, and tumor necrosis factor (TNF)-**α**, RANTES, Eotaxin, and MIG in the lysate were measured using a Bio-Plex multiplex bead array kit (Bio-Rad, Hercules, CA). The Bio-Plex assay was performed in accordance with the manufacturer’s instructions.

### RNA isolation and microarray processing

Bronchoalveolar lavages (BAL) were collected two weeks prior to challenge and at days 3, 7, and 10 post infection from animals in both the control and vaccinated group. Total RNA was isolated from BAL using Trizol and fluorescently labeled probes were generated from each sample using Agilent one-color LowInput Quick Amp Labeling Kit (Agilent Technologies). Individual cRNA samples were hybridized to oligonucleotide microarrays for gene expression profiling using Macaque 8x60K Custom Microarray Kit (G4102A; Agilent Technologies). RNA isolated from pre-challenge animals served as an uninfected reference.

The primary transcriptomic data was extracted and quantile normalized using the ‘normalizeBetweenArrays’ method available in ‘limma’ package of the R suite as previously described [80]. Differential expression of mock-vaccinated and vaccinated animals was determined by comparing the average gene expression of influenza virus-challenged animals to the average expression prior to challenge for each group applying a linear model fit using the ‘limma’ package. Criteria for differential expression were an absolute fold-change of 1.5 and an adjusted p-value of <0.05, calculated by Benjamini-Hochberg correction [81]. The microarray expression data generated in this study is available via the following accession identifier on the NCBI-GEO database: (GSE77824).

### Functional analysis of gene expression

Functional analysis of differentially expressed (DE) genes was performed using Ingenuity Pathway Analysis (IPA, Ingenuity Systems). IPA functional enrichment was calculated using a right-tailed Fisher’s exact test with a threshold of significance set at a p-value of 0.05 and presented as–log10 p-values. Enrichment of Diseases and Biological Functions were based on activation Z-scores with an absolute value >2 using IPA. The primary purpose of an activation z-scores is to infer whether a biological function is increased or decreased based on the gene expression changes[82].

### Statistical analyses

All of the analyses were performed using Graphpad Prism version 5.01. A Student's t test (to compare two samples) and analysis of variance (ANOVA) (to compare multiple samples) were used for statistical analysis. A Mann-Whitney U test was used to compare immune responses and viral loads between vaccinated and control groups in the macaque studies. A Spearman-rank correlation test was used to analyze correlations between immune responses and viral loads. A p value of <0.05 was considered to be significant. The use of N = 8 for immunogenicity and N = 5 for efficacy studies is based on prior experience, goals in comparing the groups, and power analysis using Mann Whitney U test to compare expected outcomes between groups. Group sizes were determined based efficacy. Based on power analysis, a minimum of N = 5 per group is expected to provide 76.5–93.1% power to show a 2-fold difference in immunogenicity or viral load measurements in blood or mucosal specimens between treatment groups and untreated controls. This assumes the standard deviation in mean responses for each measure range from 35–55%.

## Supporting information

S1 Checklist The ARRIVE guidelines checkist. Animal Research: Reporting in Vivo Experiments.
(PDF)

S1 Fig Intracellular cyotkine staining (ICS) gating scheme. Displayed is a representative gating scheme from peripheral blood mononuclear cells (PBMC’s) to quantify CD4^+^ and CD8^+^ frequencies of CD107ab, IFN-**γ**, TNF-**α**, and IL-2 in unstimulated (DMSO) and polyclonal *Staphylococcal* enterotoxin B (SEB) stimulated cells.
(TIF)

S2 Fig Total magnitudes of influenza specific T cells in the blood were similar in vaccinated and control macaques. Shown are the frequencies of influenza specific T cellular immune responses, at 11 days post-infection, from peripheral blood mononuclear cells (PBMC’s) including CD4^+^ and CD8^+^ T cell frequencies of CD107ab, IFN-**γ**, TNF-**α**, and IL-2 following stimulation with peptides above background levels.
(TIF)

S3 Fig Vaccinated macaques had greater frequencies cyotkine secreting HA and NP specific T-cells. Shown are the frequencies of influenza (HA and NP) specific T cellular immune responses, at either Week 14 or Week 16+4, from peripheral blood mononuclear cells (PBMC’s) including CD4^+^ and CD8^+^ T cell frequencies with various combinations of IFN-**γ**, TNF-**α**, and IL-2 following stimulation with peptides above background levels. P values are the results of non-parametric Mann-Whitney tests.
(TIF)

S4 Fig Lung immunophenotyping gating scheme. Representative flow cytometry staining of bronchioalveolar lavage (BAL) derived cells for Macrophage (Mφ), B cells as well as CD4^+^ and CD8^+^ T-cell enumeration.
(TIF)

S1 Table Antibody isotype, cojugate and conentration for Intracellular Cytokine Staining (ICS). Displayed is the panel used for assesing influenza peptide specific responses in the PBMC by ICS, indicating the laser utilized, marker and conjugate, clone, catalong number, vendor, and dilution.
(TIF)

S2 Table Antibody isotype, cojugate and conentration for lung immunophenotyping. Displayed is the panel used for assesing bronchioalveolar lavage (BAL) derived cells for Macrophage (M**Φ**), B cells as well as CD4^+^ and CD8^+^ T-cell enumeration.
(TIF)
