# Supplementary figures and images for: Multigenic DNA vaccine induces protective cross-reactive T cell responses against heterologous influenza virus in nonhuman primates
Source: PLoS One. 2017 Dec 21;12(12):e0189780. doi: 10.1371/journal.pone.0189780 (PMC5739435; doi:10.1371/journal.pone.0189780)

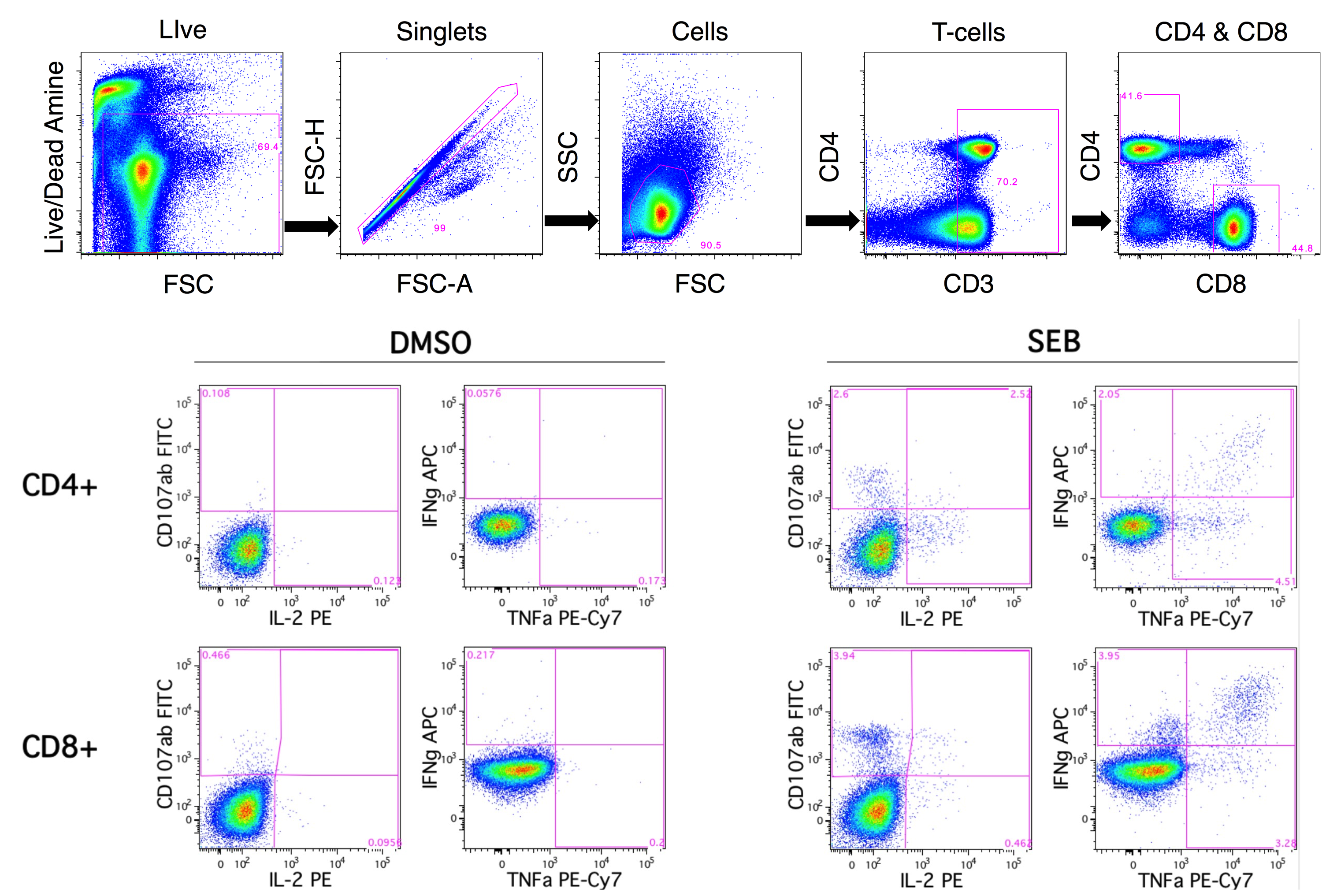

Supplement: S1 Fig — Displayed is a representative gating scheme from peripheral blood mononuclear cells (PBMC’s) to quantify CD4+ and CD8+ frequencies of CD107ab, IFN-γ, TNF-α, and IL-2 in unstimulated (DMSO) and polyclonal Staphylococcal enterotoxin B (SEB) stimulated cells. (TIF) [file pone.0189780.s002.tif]

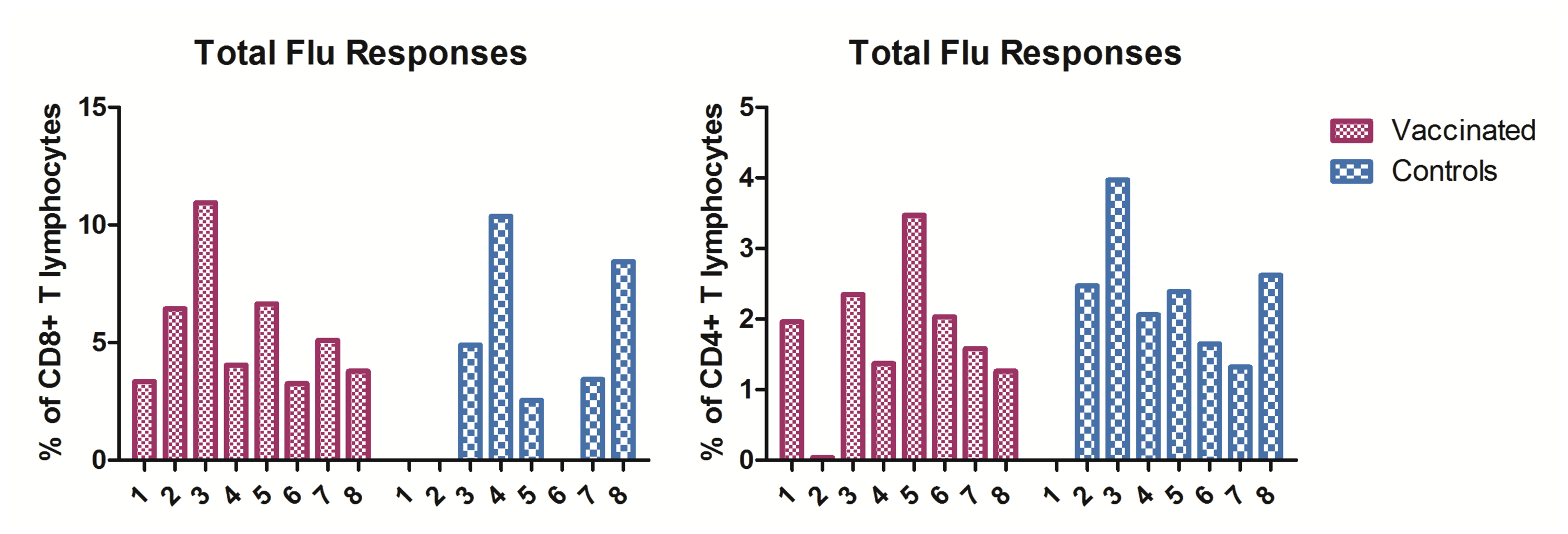

Supplement: S2 Fig — Shown are the frequencies of influenza specific T cellular immune responses, at 11 days post-infection, from peripheral blood mononuclear cells (PBMC’s) including CD4+ and CD8+ T cell frequencies of CD107ab, IFN-γ, TNF-α, and IL-2 following stimulation with peptides above background levels. (TIF) [file pone.0189780.s003.tif]

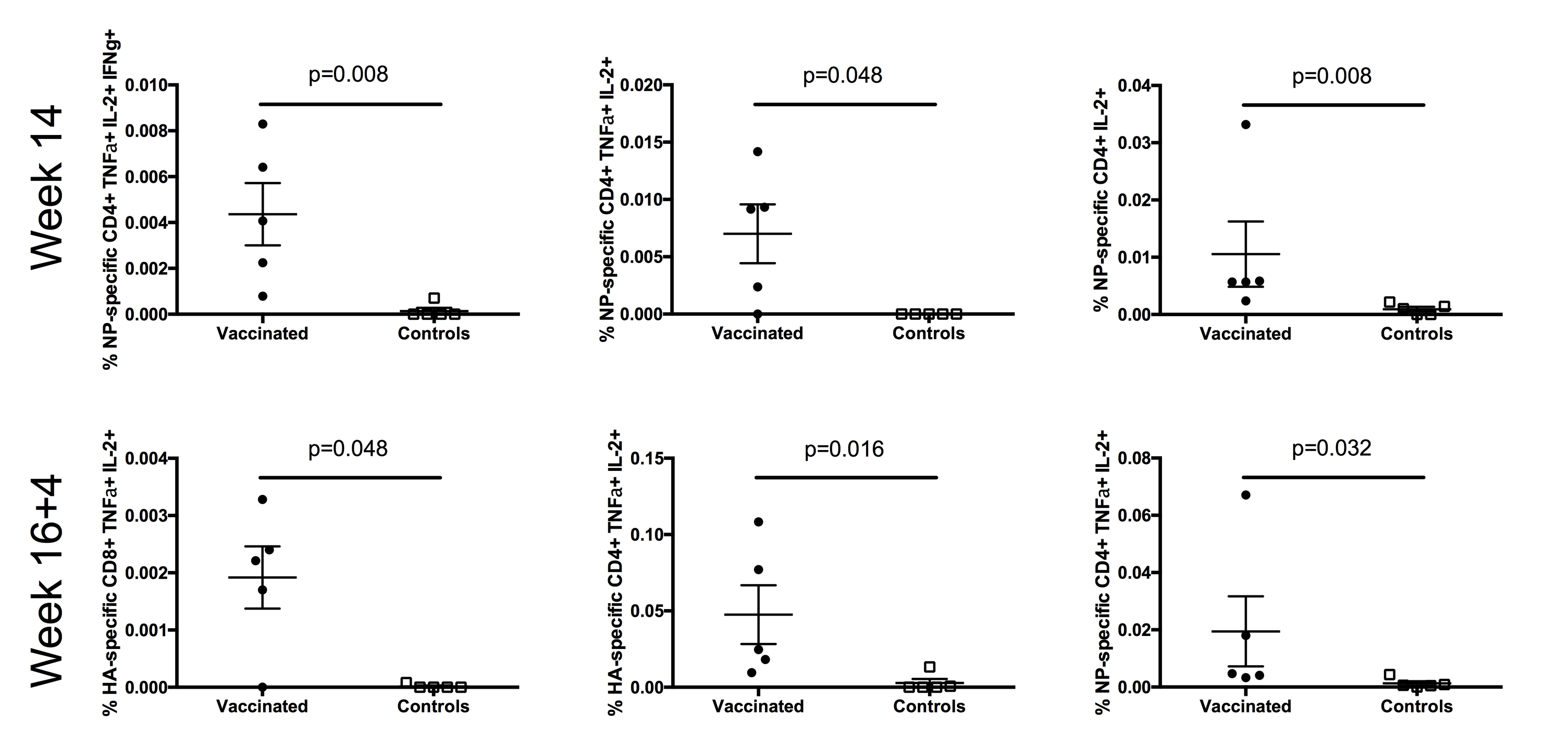

Supplement: S3 Fig — Shown are the frequencies of influenza (HA and NP) specific T cellular immune responses, at either Week 14 or Week 16+4, from peripheral blood mononuclear cells (PBMC’s) including CD4+ and CD8+ T cell frequencies with various combinations of IFN-γ, TNF-α, and IL-2 following stimulation with peptides above background levels. P values are the results of non-parametric Mann-Whitney tests. (TIF) [file pone.0189780.s004.tif]

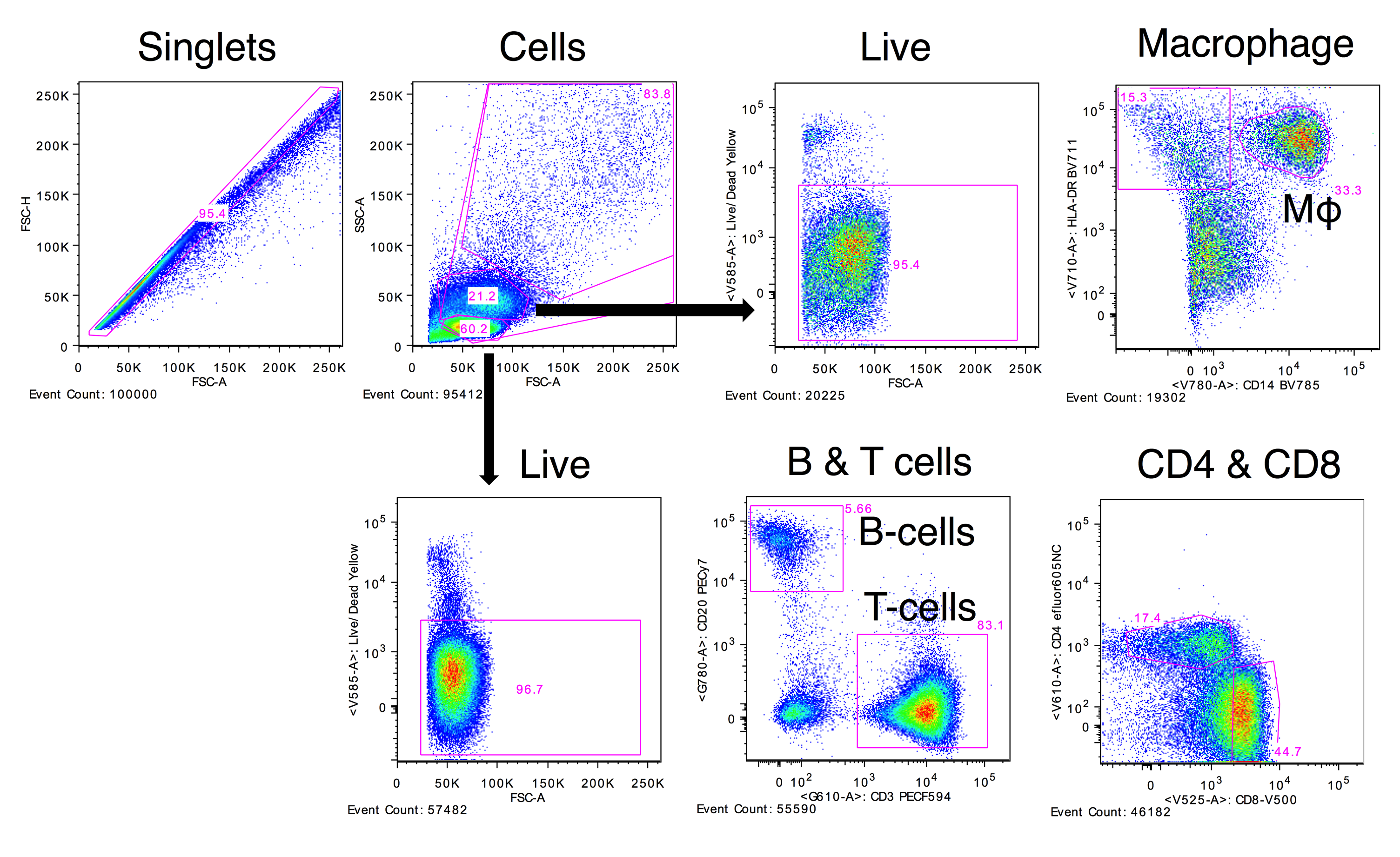

Supplement: S4 Fig — Representative flow cytometry staining of bronchioalveolar lavage (BAL) derived cells for Macrophage (Mφ), B cells as well as CD4+ and CD8+ T-cell enumeration. (TIF) [file pone.0189780.s005.tif]

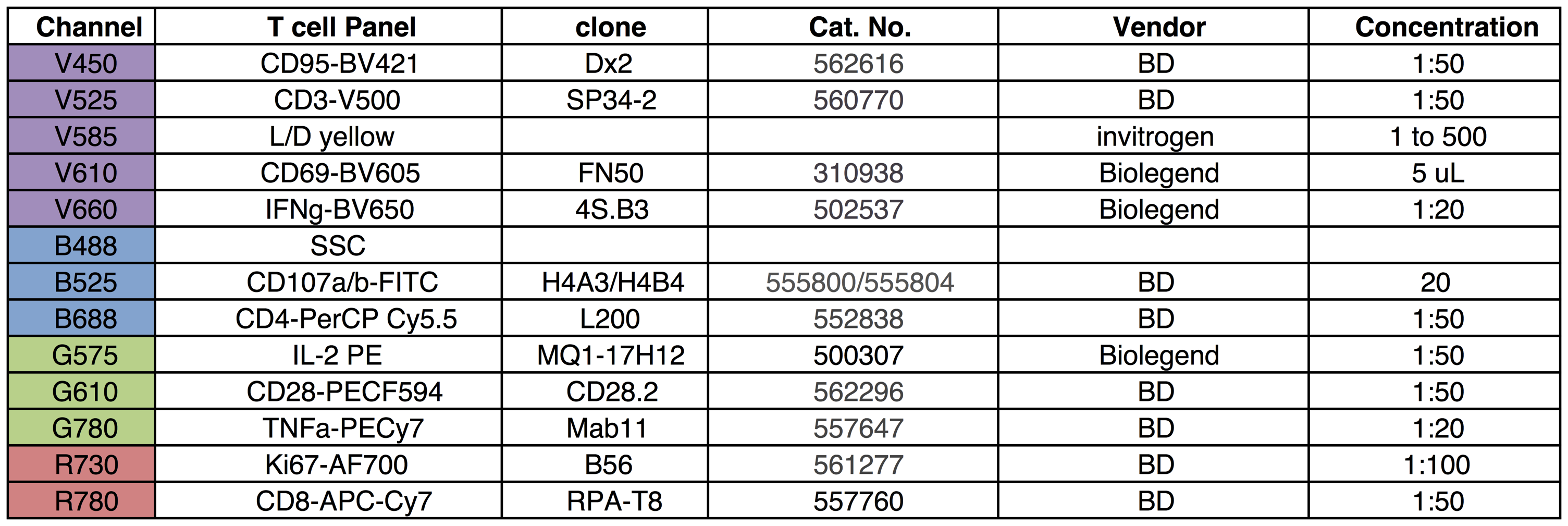

Supplement: S1 Table — Displayed is the panel used for assesing influenza peptide specific responses in the PBMC by ICS, indicating the laser utilized, marker and conjugate, clone, catalong number, vendor, and dilution. (TIF) [file pone.0189780.s006.tif]

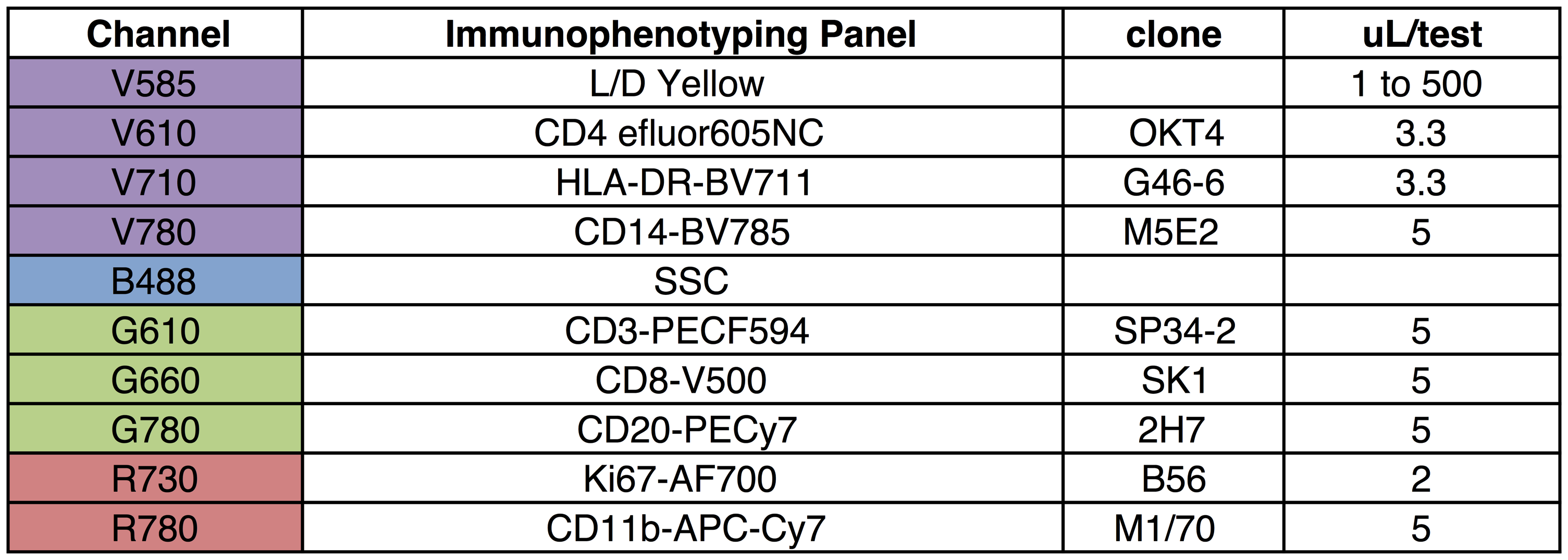

Supplement: S2 Table — Displayed is the panel used for assesing bronchioalveolar lavage (BAL) derived cells for Macrophage (MΦ), B cells as well as CD4+ and CD8+ T-cell enumeration. (TIF) [file pone.0189780.s007.tif]
